# The Classic Land of Suicide

**Published:** 1861-04

**Authors:** 


### Art. II.—THE CLASSIC LAND OF SUICIDE.

There was a time when it was customary, both at home and abroad (and we are not quite certain that the custom as yet has quite died out among ourselves), to look upon England as "the classic land of suicide." "La terre classique du suicide," was a phrase of our immediate neighbours across the straits as applied to this country; "O Britain, infamous for suicide!" sang one of our most popular poets.* A better knowledge of the subject has, however, pretty clearly established that, in this respect, we belied ourselves, and suffered others to belie us. With that happy facility for parading our shortcomings which is so incomprehensible to other nations, we, before the era of statistics, succeeded in imposing as well upon our neighbours as ourselves the belief that suicide was in an especial manner a bane of this kingdom. We now know that there was no sufficient ground for this belief before statistical returns could be appealed to, and that since these have existed, the unenviable pre-eminence of being the chief haunt of suicide can no longer be assigned to England.†

But we should gain little by getting rid of this stigma, if, to the poetical literature of this kingdom is to be attributed, as certain continental writers will have it, a primary influence in the development of that æsthetical treatment of suicide which has been one of the most curious phases of the literary history of the past eighty years.

According to Goethe, that life-weariness so productive of suicide, which was widely prevalent among the German youth, and indeed the youth of other continental nations, towards the termination of the last century, would not have been so decidedly manifested had it not been for the action of an outward cause. Such a cause existed for them, he states, in English literature, "especially the poetical part, the great beauties of which are accompanied by an earnest melancholy which it communicates to every one who occupies himself with it. . . . . One finds in it throughout a great, apt understanding, well practised in the world, a deep, tender heart, an excellent wit, an impassioned action,—the very noblest qualities which can be praised in an intellectual and cultivated man: but all this put together still makes no poet. Fine poetry announces itself thus, that, as a worldly gospel, it can by internal cheerfulness and external

---

* Young, *Night Thoughts;* Night v. l. 442.

† See Brierre du Boismont, *Du Suicide*, p. 368; *Journal of Psychological Medicine*, vol. xii. p. 216; Marc d'Espine, *Statistique Mortuaire Comparée*, pp. 96—101.

comfort free us from the earthly burdens which press upon us. Like an air-balloon, it lifts us, together with the ballast which is attached to us, into higher regions, and lets the confused labyrinths of the earth lie developed before us as in a bird's-eye view. The most lively, as well as the most serious works, have the same aim of moderating both pleasure and pain by a felicitous intellectual form. Let us only in this spirit consider the majority of the English poems, chiefly morally didactic, and on the average they will only show us a gloomy weariness of life. Not only Young's *Night Thoughts,* where this theme is pre-eminently worked out, but even the other contemplative poems, stray, before one is aware of it, into this dismal region, where the understanding is presented with a problem which it cannot solve, since even religion, much as it can always construct for itself, leaves it in the lurch."

In further illustration of this position Goethe refers to Milton's *Allegro,* in which gloom has to be scared away in "vehement verses" before even moderate pleasure can be attained; and to the *Deserted Village,* in which the cheerful Goldsmith, losing himself in elegiac feelings, "as charmingly as sadly exhibits to us a lost Paradise which his *Traveller* seeks over the whole earth." Giving us credit for at least some cheerful poetry, he adds:—

"Enough: those serious poems, undermining human nature, which in general terms have been mentioned above, were the favourites which we sought out before all others, one seeking, according to his disposition, the higher elegiac melancholy, another the heavy oppressive despair, which gives up everything. Strangely enough, our father and instructor, Shakspeare, who so well knew how to diffuse a pure cheerfulness, strengthened our feeling of dissatisfaction. Hamlet and his soliloquies were spectres which haunted all the young minds. The chief passages every one knew by heart and word to recite, and everybody fancied he had a right to be just as melancholy as the Prince of Denmark, though he had seen no ghost, and had no royal father to avenge."

Finally, *Ossian* had charmed them "even to the *Ultima Thule.*"*

Again, Brierre du Boismont tells us that Shakspeare is the source of the æsthetical literature of suicide of our own times. "Thus," he writes, "Hamlet cries:—

"To die;—to sleep;
To sleep! perchance to dream;—ay, there's the rub.

---

* *Autobiography,* translated by J. Oxenford; Bohn. Vol. i., pp. 504—7.

Already in this poet are found the principal features which characterize that literature: the dread of death and doubt of the future."* Pierre Leroux also writes in his observations upon the poetry of our epoch—" Shakspeare leads the choir of poets, Shakspeare who had conceived doubt in his breast long before philosophy. Werther and Faust, Childe Harold and Don Juan follow the shade of Hamlet, followed themselves by a multitude of despairing and lamenting phantoms, who all seem to have read the terrible legend over hell's gates, *Lasciate la speranza*."†

But, truly, it would be no easy task to trace in what manner the "earnest melancholy" which tinctures the writings of our great poets could, as Goethe asserts, become the fostering cause of that self-cultivated, self-indulged life-weariness, the prime factors of which were irreligion and scepticism, the prime result immorality, the most revolting one suicide. In no respect would the task be lighter to search for and discover in Shakspeare the source of our modern æstheticism of suicide; or to show that Werther was directly descended from Hamlet.

It is customary when looking upon a muddy stream to attribute the muddiness to the nature of the banks between which the water flows; or when gazing through a fog upon the distorted outline of a coast, to ascribe the distortion to the fog. Certainly it may be said that without the water there would be no turbid stream; without the coast no distorted coast-line. And so of the influence exercised by the poetic literature of this land upon the "Young German Mind" in Goethe's time. It may have been, as he would have us believe, that but for this literature the life-weariness which then infected the youth of his country would never have become so greatly developed as it did become. Yet who would now recognise such a source in the noxious literature which is the most enduring expression of that life-weariness, and of which *Werther* is the first-begotten and type? The source may be there, but whence the pollution? The coast may still appear above the horizon, but whence its fantastic contour? This is to be sought in the then mental state of the German youth, which, as a crumbling bank, or a thick haze, muddied or distorted whatever passed over or was seen through it.

That profound religious element which permeates and governs the grave tone of the *Deserted Village* and *Traveller*, and which is the great motive and key of the *Night Thoughts*, is dismissed by Goethe as only worthy of the brief consideration of a sneer; and that seriousness which in a Christian is presumed to be the

---

* *Du Suicide*, p. 173.

† *Werther*, edited by P. Leroux, with a preface by George Sand, p. xxviii; and *Revue Encyclopédique*, 1851; De la Poésie de notre époque.

best safeguard and best fosterer of morality, is looked upon by him as "undermining human nature." This is true to the dominant intellectual spirit of the time of which Goethe wrote, when, as Carlyle forcibly sums up,—"Whatever belonged to the finer nature of man had withered under the Harmattan breath of Doubt, or passed away in the conflagration of open Infidelity; and now, where the Tree of Life once bloomed and brought fruit of goodliest savour, there was only barrenness and desolation. To such as could find sufficient interest in the day-labour and day-wages of earthly existence; in the resources of the five bodily senses, and of Vanity, the only mental sense which yet flourished, which flourished, indeed, with gigantic vigour, matters were still not so bad. . . . . But to men afflicted with the 'malady of thought,' some devoutness of temper was an inevitable heritage; to such the noisy forum of the world could appear but an empty, altogether insufficient concern; and the whole scene of life had become hopeless enough."* These men, thus afflicted, accustomed themselves (to use a phrase of Jaques), "to suck melancholy out of a song, as a weasel sucks eggs;" and they made the older poetical literature of this country minister to this passion, by straining off that high moral feeling which gives significance to the sadness that characterizes the greater bulk of it, and retaining the mere dregs of a meaningless melancholy. To minds thus tinctured Ossian would fittingly "take the place of Homer in the heart and imagination."†

If we would more thoroughly understand the extent to which the nature of the melancholy which so largely prevails in the older poetical literature of this country, as well as the signification ordinarily attached to the word by our most trusted writers, have been misconceived by Goethe, and from the causes we have assigned, illustrations are readily found.

"That calm and elegant satisfaction," writes Steele, "which the vulgar call melancholy, is the true and proper delight of men of knowledge and virtue. What we take for diversion, which is a kind of forgetting ourselves, is but a mean way of entertainment, in comparison of that which is considering, knowing, and enjoying ourselves. The pleasures of ordinary people are in their passions; but the seat of this delight is in the reason and understanding. Such a frame of mind raises that sweet enthusiasm, which warms the imagination at the sight of every work of nature, and turns all around you into picture and landscape."‡

Again, and still more to our purpose, Mackenzie writes, at a period when *Wertherism* was in progress of development,—

---

* *Miscellanies:* Art. Goethe.

† *Werther*, Octr. 12. ‡ *Tatler*, No. 89, Nov. 3, 1709.

"You say truly, in one of your late papers [referring to a previous number of the *Lounger*], that poetry is almost extinguished among us: it is one of my old-fashioned propensities to be fond of poetry, to be delighted with its descriptions, to be affected by its sentiments. I find in genuine poetry a sort of opening to the feelings of my mind, to which my own expression could not give vent; I see, in its descriptions, a picture more lively and better composed than my own less distinct and less vivid ideas of the objects around me could furnish. It is with such impressions that I read the following lines of Thomson's Autumn, introductive of the solemn and beautiful apostrophe to philosophic melancholy:—

"But see the fading many-coloured woods,
Shade deepening over shade, the country round
Imbrown; a crowded umbrage, dusk and dun,
Of every hue, from wan declining green
To sooty dark. These now the lonesome Muse,
Low-whispering, lead into their leaf-strewn walks,
And give the season in its latest view.
Meantime, light-shadowing all, a sober calm
Fleeces unbounded ether; whose least wave
Stands tremulous, uncertain where to turn
The gentle current; while illumined wide,
The dewy-skirted clouds imbibe the sun,
And through their lucid veil his soften'd force
Shed o'er the peaceful world. This is the time
For those whom wisdom and whom nature charm
To steal themselves from the degenerate crowd,
And soar above this little scene of things;
To tread low-thoughted vice beneath their feet;
To soothe the throbbing passions into peace,
And woo lone quiet in her silent walks.

"About this time three years, sir, I had the misfortune to lose a daughter, the last survivor of my family, whom her mother, dying at her birth, left a legacy to my tenderness, who closed a life of the most exemplary goodness, of the most tender filial duty, of the warmest benevolence, of the most exalted piety, by a very gradual but not unperceived decay. When I think on the returning season of this calamity, when I see the last fading flowers of Autumn, which my Harriet used to gather with a kind of sympathetic sadness, and hear the small chirping note of the flocking linnets, which she used to make me observe as the elegy of the year; when I have drawn her picture in the midst of this rural scenery, and then reflect on her many virtues and accomplishments, on her early and increasing attentions to myself, her gentle and winning manners to every one around her; when I remember her resignation during the progress of her disorder, her unshaken and sublime piety in its latest stages; when these recollections fill my mind, in conjunction with the drooping images of the season, and the sense of my own waning period of life; I feel a mixture of sadness and of composure, of humility and elevation of spirit, which I think, sir, a man would ill exchange for any degree of unfeeling prudence or of worldly wisdom and indifference."*

This fragment from the *Lounger* serves as the best interpretation to be attached to Steele's observations, and at the same time it teaches us in the happiest manner the meaning to be assigned to the melancholy which pervades the writings of our standard poets, and the great contrast between this meaning and the one which Goethe sought to affix to that emotional state as exemplified by their works. This melancholy, as is so exquisitely shown by Mackenzie's reflections on the death of his daughter, is but the reflex of a self-communing habit of thought which is accustomed to look upon all things as having a high moral significance. Hence it is that the moral element, inspired by religious belief, alone gives significance and vitality to the sombre thoughts of our great poets; and to dissociate this element from these thoughts, as Goethe does, is to render them utterly void of meaning.

Thomson, in his apostrophe to Philosophic Melancholy,† has so fully expressed the true *English* sense of the term, that it would be folly to attempt to convey this in any other language. He describes the approach of melancholy as being declared by the sudden-starting tear, the glowing cheek, the mild dejected air, the softened feature, and the beating heart, "pierced deep with many a virtuous pang." A sacred influence is breathed over the soul, and the imagination inflaming, infuses every tenderness through the breast, and exalts the swelling thought far beyond the dim earth.

> "Ten thousand thousand fleet ideas, such
> As never mingled with the vulgar dream,
> Crowd fast into the mind's creative eye.
> As fast the correspondent passions rise,
> As varied and as high: Devotion, raised
> To rapture and divine astonishment
> The love of nature unconfined, and, chief,
> Of human race: the large ambitious wish
> To make them blest; the sigh for suffering worth
> Lost in obscurity; the noble scorn
> Of tyrant-pride; the fearless great resolve;
> The wonder which the dying patriot draws,
> Inspiring glory through remotest time;
> Th' awaken'd throb for virtue and for fame;
> The sympathies of love and friendship dear;
> With all the social offspring of the heart."

---

* *The Lounger*, No. 93, Nov. 11, 1786.

† Written A.D. 1730.

Such is a portraiture of the self-indulged melancholy which the English poet derives from nature; but what traces of it can we detect in *Werther?* Solely the tearfulness, the dejection, the inflamed imagination, and the host of fleeting ideas, but unballasted by any virtuous pang, by any high-souled tenderness, by any of that nobility of thought which religion pre-eminently gives, and which is shown in the abnegation of self, in that large-heartedness which ever seeks to succour and advance the human race, in a lofty patriotism, in a noble struggle for virtue and fame, or in the holy ties of domestic life.

What is absent in *Werther* is also absent from Goethe's estimate of the influence of English poetical literature in the production of *Wertherism*, that is to say, that whatever there is of excellence in the melancholy of our great poets is cast aside as worthless, and the mere unmeaning fact of gloominess retained.

And now to turn from the general question to the special illustration, and compare briefly Hamlet, the assumed prototype of the leading characters found in the modern æsthetical literature of suicide, with Werther, the earliest produced and chief of them.

Hamlet is depicted, first, as woe-begone at the loss of a much-loved father, and with his soul wrung by the incestuous marriage of his mother.

> "Within a month,—
> Let me not think on't;—Frailty, thy name is woman!—
> A little month; or ere those shoes were old,
> With which she follow'd my poor father's body,
> Like Niobe, all tears;—why she, even she,—
> O heaven! a beast, that wants discourse of reason,
> Would have mourn'd longer,—married with my uncle,
> My father's brother; but no more like my father
> Than I to Hercules: Within a month;
> Ere yet the salt of most unrighteous tears
> Had left the flushing in her galled eyes,
> She married:—O most wicked speed, to post
> With such dexterity to incestuous sheets!
> It is not, nor it cannot come to, good;
> But break, my heart: for I must hold my tongue!"

Hamlet, thus woe-begone, and compelled to keep his griefs hid within his own breast, suffers an aspiration of regret to pass his lips that the Everlasting had "fix'd his canon 'gainst self-slaughter." And, again, when crushed by the horrible secret, brought to him from the nether world, of his father's murder, and the equally horrible duty imposed upon him by his father's ghost of murdering (for even the latitudinarian notions of revenge indulged in Hamlet's supposed time would admit no softer word for the act) his uncle, his mother's husband, he is represented as once more resorting to suicide as a means of escaping from the "sea of troubles" which had overwhelmed him. But as his religious notions heretofore, so his reason, or as it is the custom to say, his philosophy, now rebuts, and that quickly, the notion. There is no tampering with the doctrine inculcated by the Church that suicide is a damnable sin, and by the State that it is a crime; there is no attempt to fritter away under paradoxes and conceits the, to reason, inscrutable question—

> "To die; to sleep;
> To sleep! perchance to dream; ay, there's the rub:
> For in that sleep of death what dreams may come,
> When we have shuffled off this mortal coil:"

no; the mind at once pauses, apprehending its own helplessness in unravelling the mighty problem, and the conscience haunted by the dread of "something after death," again causes the idea of suicide to be cast aside.

And yet it might be supposed that Hamlet, a scholar, hankering for death as the easiest means of escaping the grievous troubles which had beset him, would but too readily have sought counsel from the writings of those ancient philosophers who had justified suicide, when either bodily or mental suffering taxed our endurance. The name of the greatest of these, Seneca,—one who like Werther, always held it as a consolation that it was at his will to leave the world when he liked,* and who had represented Deity as teaching us that "if we choose not to fight against evils, we may fly from them: therefore, of all things which he had made necessary for us, he made none so easy as to die,"†—had but a little while before been in the mouth of Polonius when addressing Hamlet.‡ But the latter never attempts thus to pander to his feelings. Whence comes this? It is evident that the belief that suicide was an offence against the laws of God, which had governed Hamlet's thoughts when he first reverted to the subject, also controlled them when he again returns to it. It is this conviction, unexpressed, but which together with that thorough belief in the existence of a heaven and a hell so fully manifested or implied in the ghost scene, the scene where the King is praying, and elsewhere in the play, which curbs Hamlet's philosophical speculations on death and gives them that peculiar character in the great soliloquy, "To be, or not to be," which some commentators have thought to be inconsistent with the scenes referred to. In fact, there is no incidental feature in the delineation of Hamlet of greater interest than the mode in which the doctrines of the Christian Church are shown to hold in

* Ep. lxx. † *De Providentia.* ‡ Act ii. sc. 2.

check the propensity to suicide—a feature characteristic of the history of suicide among Christian nations at the time in which the action of the play may be considered as having taken place. It remained for our own time, and for the young German mind first of all in the character of Werther, to show that the truths of Christianity could be pleasingly dovetailed into the pagan doctrines of suicide.

Werther is represented as having recourse to suicide in order to escape the mental misery occasioned by an unrestrained and adulterous passion for the wife of a friend. He has no trouble but what is of his own creation, none but what is dependent upon the deprivation or lack of some sensuous pleasure—refined it may be (as the world goes), but sensuous nevertheless. He hugs these troubles and cherishes them as holy things; but "hemmed in as he is, he [like the ancient Stoics] ever keeps in his heart the sweet feeling of freedom, and that this dungeon *can be left when he likes.*"* He treats suicide as a legitimate thing, morally, and buries the iniquity and folly of the deed beneath a heap of wretched paradoxes. He entertains no other ideas of morality than those which are involved in its conventional practice; he adopts religious beliefs only so far as they may be modified so as to foster his peculiar vices; and having thus modified them, he advances his great and final woe, the uncontrolled and ungratified passion for his friend's wife, as a crown of martyrdom and a sure ground of beatitude hereafter!

"Everything passes away, but a whole eternity could not extinguish the living flame which was yesterday kindled by your lips, and which now burns within me. She loves me! these arms have encircled her waist, these lips have trembled upon hers. She is mine! Yes, Charlotte, you are mine for ever!

"And what do they mean by saying Albert is your husband? He may be so for this world: in this world it is a sin to woo you—to wish to tear you from his embrace. Yes, it is a crime, and I suffer the punishment; but I have enjoyed the full delight of my sin. I have inhaled a balm that has revived my soul. From this hour you are mine; yes, Charlotte, you are mine! I go before you. I go to my Father, and to your Father. I will pour out my sorrows before Him, and He will give me comfort till you arrive. Then will I fly to meet you. I will claim you, and remain in your eternal embrace in the presence of the Almighty.

"I do not dream, I do not rave. Drawing nearer to the grave, my perceptions become clearer. We shall exist; we shall see each other again; we shall behold your mother; I shall behold her, and expose to her my inmost heart. Your mother—your image!"†

Hamlet and Werther:—is not this another reading of Hyperion

* *Letter*, 22nd May.

† *Werther*, Bohn's Ed., p. 348.

to a satyr? The one at all times curbed more or less, directly or indirectly, by the broad doctrines of Christian morality and truth, the other deliberately mutilating these doctrines and prostituting them to the service of vice and suicide, decorating the latter also with a wealth of meretricious sentiment; the one recoiling from and seeking to evade the dread task imposed upon him by Revenge for a murdered father, the other playing with the idea of murder as a fitting means of terminating his self-generated troubles. "I will die," writes Werther. "It is not despair, it is conviction that I have filled up the measure of my sufferings, that I have reached the term, and that I sacrifice myself for you. Yes, Charlotte, why should I not say it? It is necessary for one of us three to depart—it shall be Werther. Oh! my dear Charlotte! this heart, governed by rage and fury, has often conceived the horrid idea of murdering your husband—you—myself."* Hamlet and Werther must ever stand wide apart from each other; no just analysis will approximate their characters. It is insufficient to say that there can be detected in Hamlet the germ of that spirit of doubt which besets Werther and his congeners. The doubt of the former is that incidental to one whose soul has been borne down and unbraced by accumulated sorrow, and who, in the bitterness of his heart, would choose death rather than life; the doubt of the latter is the mainspring of his thoughts and actions. The explanation of Hamlet's doubt need not, nor should it, be sought in any system of philosophy or philosophical notions of his presumed time, but in the pages of Spenser,—of whom Shakspeare himself has written:—

"whose deep conceit is such,
As passing all conceit, needs no defence."

Also:—

"And I in deep delight am chiefly drown'd,
Whenas himself to singing he betakes."†

Read how Despair pleads with the Red Cross Knight:—

"Is not short payne well borne, that brings long ease,
And layes the soul to sleep, in quiet grave?
Sleep after toyle, port after stormie seas,
Ease after warre, death after life, does greatly please."

. . . . . . .

Die shall all flesh? What then must needs be donne?
Is it not better to die willinglie,
Than linger till the glas be all out ronne?
Death is the end of woes: Die soone, O Faerie's sonne."‡

---

* *Letter*, 20th Dec.
† *The Passionate Pilgrim*, Sonnet vi. "If music and sweet poetry agree," &c.
‡ *The Faerie Queene*, Bk. i. c. 9.

But when Hamlet uttered his doubts there was no Una standing by to withdraw him from the counsels of the tempter. Once before she had saved him; but now Revenge had usurped her place, and such reasons as he could urge for life served to add, not detract, from the desire for death. What, then, was left which could hold Hamlet back from suicide, but the dread of an hereafter?—in him the faint and murky, but still unextinguished reflection of that Christian element which comes to the surface throughout the play. The doubting of Hamlet is that to which all men have been liable, and to which many have yielded, in all ages of the world, when exposed to excess of mental or bodily suffering: the doubting of Werther is that which is peculiar to an extravagant philosophical scepticism, whether ancient or modern. The one is the result of the mind succumbing to a stress of grief; the other is the product of a self-satisfied and self-confident system of reasoning.

Again, it is insufficient to say that in Hamlet we have the prototype of those characters in whom action is enfeebled by exaggerated, undue mental activity. "In Hamlet we see," writes Coleridge, "an enormous intellectual activity, and a proportionate aversion to real action consequent upon it, with all its symptoms and accompanying qualities. This character Shakspeare places in circumstances under which it is obliged to act on the spur of the moment. Hamlet is brave, and careless of death; but he vacillates from sensibility and procrastinates from thought, and loses the power of action in the energy of resolve."* "The preponderance of thought and speech over action," writes Du Boismont, "in a word, feebleness, is the foundation of the fanciful and melancholy heroes of suicide."† But even were we to admit the correctness of these abstract views, it does not in any degree help us to reconcile the concrete characters of Hamlet and the Werther school, or to trace the development of the latter, even remotely, to the influence of the former. No parallelism exists whatever, either in the causes leading to the development, or in the mode of growth, or in the results of the disgust of life which is pourtrayed in the mediæval (assumed) hero and our modern heroes of suicide. In the latter we behold, as Goethe tells us he desired to set forth in the character of Werther, "that disgust which man, without being driven to necessity, feels for life."‡ "We have here," he again says, "to do with those whose life is embittered by a want of action, in the midst of the most peaceful circumstances in the world, through exaggerated demands upon themselves."§ In short, Hamlet belongs to all time, Werther to a peculiar epoch, and there is no greater resemblance between the

* *Literary Remains*, Vol. iv. p. 205. † *Du Suicide*, p. 174.

‡ *Autobiography*, Bohn, Vol. i. p. 502.

§ *Op. cit.*, Vol. i. p. 50.

two characters than that which exists (to take a simile from Bunyan's character of Self-Will, who represents in no small degree the religious phase of Wertherism) between a child that has been cast down by a blast of wind or tripped up by a stone, and defiled itself in the mire, and one who has wilfully laid down and wallowed like a boar therein.

Neither in Shakspeare, nor in the older poetical literature of England as a whole, nor, indeed, in the entire literature of this country prior to the publication of *Werther*, can be found the special characteristics of the modern æsthetical literature of suicide. Truly we had, when that book first appeared, our apologists for suicide. Suicide was also then notoriously very common among us, and the philosophical scepticism which at that time pervaded France and Germany, and which frittered away all that was vital in religion and morality, prevailed extensively in England.

Europe, indeed, at the period we are writing of, was profoundly disquieted. The influence of religion and morals over men's minds, and the authority of the Church, had been waning throughout the century, and towards its termination had become greatly enfeebled. Philosophy, in the absence of religion the chief refuge for those higher cravings of the mind which lie at the source of our moral and social habits, was herself luxuriating, and too commonly running riot in the liberty she had just fully secured from the trammels of theological dogmas and traditions. Tainted, moreover, by the prevalent unbelief, she lent herself to support and confirm it. The sensualism of Locke, then the reigning system of philosophy, had been pushed to its most extravagant lengths in fatalism, materialism, and atheism, and thus became the chief feeder of that popular scepticism under which a declension in religion or morality at all times seeks to cloak itself. Such a scepticism was the great mental characteristic of the period, and it pervaded all classes of the people. If, however, the predominant sensualistic philosophy of the time formed its main aliment, yet it also fed, in Germany at least, upon the idealism that came in its way, deducing from that system a pure pantheism—a deduction which in every way served its purpose.

Whatever had served to lend a charm to life in literature, in art, and in social life, suffered to a greater or less extent from the blighting influence of this scepticism, itself at one and the same time a result and a fostering cause of irreligion and immorality. The bonds which held society together were relaxed, and in France they, in the end, were rent asunder with such terrible vehemence, that every nation in Europe trembled to the very centre with the shock.

The evil passions of man, so largely unrestrained except by the ignoble motives of brute force and selfishness, rushed freely to the surface, and suicide (not the least among the ills which then obtained an undue prominence) was elevated to a dignity which it had probably never before possessed among Christian nations. But even suicide itself, an act which it might have been supposed admitted of no variation in degree of infamy, became more degraded than it had ever before been. Among the ancient pagan apologists and justifiers of the deed, suicide, as a rule, was only vindicated when it was had recourse to from patriotic motives, or to escape dishonour, or unmerited or involuntary suffering, the individual having previously lived a virtuous life; while he who had committed the act, to escape from the consequences of his own evil deeds or vicious habits, was branded with ignominy. At the epoch of which we are now writing, however, notwithstanding that the virtues of the ancients were aped, these were too generally made to shield the vices of the moderns. Suicide was justified without reference to the causes of the deed, the gambler and the debauchee, as well as he who had been hounded to despair by misery of his own begetting, slipping out of the world at their convenience, and flattering themselves that they were emulating in so doing an ancient virtue.

Thus it came to pass that at the termination of the eighteenth century men's minds were deeply disturbed, and a quasi-philosophical scepticism, which, in whatever manner it might humour the reason left the feelings sterile, had usurped the place of religion.

In England, about the middle of the eighteenth century, "free-thinking" had received a fresh impetus from the publication of Lord Bolingbroke's posthumous works; and from the strictures of contemporary writers upon the prevalence of suicide at that time, we learn that this act was then attributed mainly to wilful extravagance and debauchery on the one hand, and to scepticism on the other.

> "Another principal cause of this frequency of suicide," says a writer in the *Connoisseur*,* "is the noble spirit of free-thinking which has diffused itself among all ranks of people. The libertine of fashion has too refined a taste to trouble himself at all about a soul or an hereafter; but the vulgar infidel is at wonderful pains to get rid of the Bible, and labours to persuade himself out of his religion. For this purpose he attends constantly at the disputant societies, where he hears a great deal about free-will, free-agency, and predestination, till at length he is convinced that he is at liberty to do as he pleases, lays his misfortunes to the charge of Providence, and comforts himself that he was inevitably destined to be tied up in his own garters."

The same writer satirically suggests that "if this madness (of suicide) should grow more and more epidemical," it would "be expedient to have a bill of suicide, distinct from the common bill

* No. 50, Jan. 9, 1755.

of mortality, brought in yearly: in which should be set down the number of suicides, their method of destroying themselves, and the likely causes of so doing." He believes that few would be found martyrs to the weather, and he adds the following significant sketch of a bill:—

"*A Bill of Suicide for the year —*

Of Newmarket Races,—
Of kept Mistresses,—
Of Electioneering,—
Of Lotteries,—
Of Gambling,—
Of French Wines, French Cooks, &c., &c.,—
Of Chinese Temples,—
Of a Country Seat,—
Of a Town House,—
Of Fortune Hunting,—
Of a Tour through France and Italy,—
Of Lord Bolingbrooke, &c., &c.,—
Of the Robin Hood Society,—
Of an Equipage,—
Of a Dog-Kennel,—
Of Covent-Garden,—
Of Plays, Operas, Concerts, Masquerades, Routs, Drums, &c.,—
Of keeping the best Company."*

From this period the progress of popular scepticism in England followed pretty much the same course as that of France and Germany, and with very similar results, intellectually and morally. About the termination of the century we find, also, the prevalence of suicide assigned to the existence of "melancholy" among us, the term being evidently used to convey the notion of that emotional state which Goethe endeavoured to depict in the character of Werther. A writer in the *Looker-On*† discusses, in two very interesting essays, the question of melancholy in connexion with suicide. He describes melancholy as being "among those modifications of the human character, which wait the fecundating efficacy of social refinement, ere they break out in all their diversities of shade and colouring." He asks, "how it should come to pass that an addiction to melancholy is more common among my countrymen than other Europeans." This, he conceives, is to be sought in moral, not in physical causes:

"Blame not thy clime, nor chide the distant sun;
The sun is innocent, thy clime absolved:
Immoral climes kind nature never made."‡

"If, therefore," he writes, "in our search after the grounds of this melancholy, we look no farther than the mind it inhabits, what abundant sources of secret sorrow, what a laboratory of pains and afflictions, do we there discover! In the cruel fondness of parentage, in the early plantation of deceitful hopes, and not seldom of vicious

* See also, for bitter satires on the subject of suicide, *The World*, No. 193, Sept., 1756; and the *Gentleman's Magazine*, Vol. xxv. p. 43, 1755. The article last referred to has been ascribed to Dr. Johnson.

† Nos. 85 and 86, 1794.

‡ Young's *Night Thoughts*, Night v.

principles; in the selfish luxury which is permitted to youth, and in the barren occupations to which our manhood is surrendered; in the unripe consequence with which children are invested; and in the fastidious satiety which, in our present forcing system of culture, teaches us to spurn at simple pleasures, before even half our capacities of delight are unfolded—I read the long history of human sorrows, and see the whole mischief developed in its series of causes and effects."

The essayist next considers the influence of political freedom, and remarks:—

"I hope it may be the timorous observations of an old man, for it is, indeed, a dispiriting consideration, that as we gradually mount from slavery to freedom, as we gradually draw towards the state of society most honourable to our natures, and most favourable to our natural search after knowledge and improvement, the melancholy of our mind increases and new shapes of inward sorrow are tacitly blended with our triumphs."

In another paragraph, we read:—

"But of all the sorrows whence arise that melancholy which ripens with our age, there are none so prolific as the neglect, in those on whom youth depends, of placing before them such objects and amusements as are durable, and last beyond the date of short-lived juvenility" . . . [not, however, overlooking the natural sportiveness of children].

In his observations on suicide the essayist says:—

"I am persuaded there never has existed a man brought up by his sorrows to the act of suicide, in whose history, could we get the truth concerning him, we should not . . . . find a gross principle of vanity at the bottom, a tissue of proud assumptions and expectations, and those for the greater part the result of parental indulgence and the deceitful promises of early adulation."

Clearly this writer is dealing with a crude Wertherism, and the tone of the two essays to which we have referred, throughout shows that the author, although probably unwittingly, was tainted by the peculiar disgust of the time in which he wrote, and that the "melancholy" of which he sought to investigate the causes, was of that particular form out of which Wertherism arose.

It was with this melancholy or disgust of life, a product of that mental and moral state of society which we have attempted briefly to sketch, and which existed more or less in this country and among the Christian nations of Europe, that Werther chimed in when given to the world, and which then, for the first time, received a full and definite expression. But this was not the sole, or even the chief secret of the amazing influence exercised by the book, and of the avidity with which it was everywhere seized upon. Hitherto, that vague, dreamy, objectless disquietude and depression of mind, of which Werther subsequently became the type, had in no respect been more repulsive than in their seemingly utter dissociation from all the better feelings of humanity: these had hopelessly withered away beneath their blighting influence. But the genius of Goethe effected a magical transformation. If, on the one hand, he had truly given voice to the wailings of the restless and melancholy spirits of the epoch, on the other, he had clothed all that was revolting, all that was contemptible, all that was barren and worthless of their peculiarities, in a delicate and finely-wrought veil of æstheticism, which gave to the character of Werther an aspect of being linked to the holier feelings of our nature, by many and most powerful bonds. He, in fact, infused a seeming vitality of true feeling into the sterile tracts of sceptical philosophy and morality (or rather immorality), and those who were wearily traversing or were lost in the arid desert, hailed with rapture the delusive mirage. "Werter," Carlyle writes, "appeared to seize the hearts of men in all quarters of the world, and to utter for them the word they had long been waiting to hear. As usually happens, too, this same word once uttered, was soon abundantly repeated; spoken in all dialects, and chanted through all notes of the gamut, till the sound of it had grown a weariness rather than a pleasure. Sceptical sentimentality, view-hunting, love, friendship, suicide, and desperation, became the staple of literary ware; and though the epidemic, after a long course of years, subsided in Germany, it re-appeared with various modifications in other countries, and everywhere abundant traces of its good and bad effects can still be discerned."*

If, as we have contended, the peculiarities of Werther cannot in any degree be traced to the literature of this country, and that that work was the first manifestation, and, in so far, the origin of the modern æsthetical literature of suicide, it cannot be denied that among us arose the most powerful of the race of sentimentalists, of whom Werther was the forerunner. Of these Byron was undoubtedly the greatest;† but notwithstanding this, Wer-

---

* *Op. cit.*, Art. Goethe.

† "—— Nous n' hésitons pas à donner à Byron la supériorité sur Goethe, comme poète *caractéristique* de l'époque ; car nous trouvions dans Byron, pour employer une expression même de ce poëte, *une plus grande vitalité du poison.*"

> There is a very life in our despair,
> *Vitality of poison ;* a quick root
> Which feeds these deadly branches.— *C. Harold.*

"Byron, par la nature particulière de son génie, par l'influence immense qu'il a exercée, par la franchise avec laquelle il a accepté ce rôle de doute et d'ironie, d'enthousiasme et de spleen, d'espoir sans bornes et de désolation, réservé à la poésie de notre temps, méritera peut-être de la posterité de donner son nom à cette période de l'art: en tout cas, ses contemporaines ont déjà commencé à lui rendre cet hommage."—Pierre Leroux : Introduction to *Werther*, pp. xix, xx.

therism proper (that is to say, the type being closely copied) was comparatively short-lived in England, and at the present day, perhaps, it is only to be found in France, where it still flourishes with considerable vigour.

It is a question of considerable interest whether there is any probability of a recrudescence, in this country, of that æstheticism of suicide which is one of the chief features of Wertherism. There is happily little in common between the moral and intellectual characteristics of the present day, and those of the period of which we have been writing. It is certain, however, that there is never wanting a leaven of that peculiar form of scepticism, which, as we have seen, constituted by far the most important of the intellectual elements which were efficient in the genesis and propagation of Wertherism. Two things in connexion with this subject are especially worthy of note in our own day; first, the wide-spread interest which has been excited by Mr. Buckle's fatalistic doctrines, as set forth in the introduction to his *History of Civilization in England*, and which are illustrated mainly by the statistical records of suicide and murder; and secondly, the fact that suicide is beginning to fare well at the hands of our artists.

Mr. Buckle asserts, concerning suicide, that all the evidence we possess points "to one great conclusion, and can leave no doubt on our minds that suicide is merely the product of the general condition of society, and that the individual felon only carries into effect what is a necessary consequence of preceding circumstances."* We read in the *Bhagavad-Ghita*, that—"The presumptuous thinks himself the author of his actions; but all his actions come from the force and from the necessary concatenation of things." This dogma, Cousin tells us, is "destructive of all liberty and all morality,"† yet Mr. Buckle's opinions are but a modern development of it.

Again: in the Royal Academy Exhibition of 1859, a painting hung upon the walls, simply described as *The Fumes of Charcoal*, and in which suicide was treated purely æsthetically.‡ In this painting two young persons, a male and a female, are represented committing suicide, by inhaling the fumes from burning charcoal. As the subject is dealt with, the artist would appear to have no other object than that of veneering the act of suicide with a perverted sentimentality. In the Exhibition of last year, conspicuously placed, was Mr. Solomon's large and powerful painting, in

* Vol. i. p. 25, 2nd Ed. For an examination of Mr. Buckle's evidence for this conclusion, and a proof of its insufficiency, see *Journal of Psychological Medicine*, Vol. xiv. p. 590.

† *Cours de l'Histoire de la Philosophie Moderne.* Par Victor Cousin. 6me Leçon.

‡ This painting is at present hanging in the picture gallery of the Crystal Palace, Sydenham.

which suicide is treated melo-dramatically. The time is daybreak; a golden-haired girl has just been fished out of the Thames, near one of the bridges, and brought to the head of the stairs by two watermen; the body is held in the arms of a motherly-looking flower-woman, and the face of the unfortunate is illuminated by the bull's-eye lantern of a kneeling policeman, a girl with a basket of early wild-flowers on the head standing by and looking pityingly on. A body of wild revellers in masquerade-costumes, crossing the bridge, come suddenly upon the sad group, and from the startled and horrified face of the first reveller, a gentleman, who has a brilliant and laughing Traviata hanging upon his arm, we learn the history and source of the suicide's fall.

Without ascribing undue weight to these indications of suicide becoming, or seeming as if it were about to become, a favourite subject with our artists, or to the fact of certain fatalistic doctrines, of which suicide is advanced as one of the principal illustrations and proofs, being received with avidity by the reading public, it is well to ask to what such things might tend. The answer to this question is best derived, first, from a history of the modern æstheticism of suicide, which we have endeavoured to sketch in this article; and secondly, from an examination of the latest manifestation of this æstheticism, as exhibited in recent French literature. This we propose to deal with hereafter. In conclusion we would repeat certain remarks that we have already made use of in reference to this subject in the past series of this *Journal*:—

"— Wherever Mr. Buckle's reasoning finds acceptance, it may be anticipated that it will lead to an unfortunate indifference to suicide in its social relations. Meriting neither praise nor blame, and uninfluenced by moral restraints, the act must be submitted to as a disagreeable necessity of every-day life, and we must accustom ourselves to it in the best way we can. And how will this be brought about? Shall we rest content to have this revolting creation of a new Frankenstein hunting its victims day by day to death among us in commonplace ghastly guise? Surely not. We shall strive to hide the most horrible features beneath a profusion of conceits; we shall fence in the pathways of the demon with a wealth of fanciful sentiment, and, it may be, we shall end as many others have done . . . . by enthroning an image of him, and worshipping it. . . . . Let us have a care. We have our present artists who find a charm in suicide; we have an apologist for the act in certainly one of the most facile and attractive historical writers of the day; and the prescriptions of both the law and the gospel in reference to it are in a great measure unheeded. This is not a bad starting-point and groundwork in favour of a reactionary movement, sympathetic of suicide, and if we do not take heed, we shall have our young men and maidens looking upon the deed as a matter of feeling, and not of morality. And so, in due time, we should come to hear the legitimacy of suicide babbled of at our fire-sides and in our workshops, while sympathy would find an outlet in song."*

---

### Art. III.—PROFESSIONAL TRICKSTERS.

* *Journal of Psychological Medicine,* Vol. xii. p. 601.

